# The Role of p53 Dysfunction in Colorectal Cancer and Its Implication for Therapy

**DOI:** 10.3390/cancers13102296

**Published:** 2021-05-11

**Authors:** Maurice Michel, Leonard Kaps, Annett Maderer, Peter R. Galle, Markus Moehler

**Affiliations:** 1 I. Department of Medicine, University Medical Center Mainz, 55131 Mainz, Germany; Maurice.Michel@unimedizin-mainz.de (M.M.); Leonard.Kaps@unimedizin-mainz.de (L.K.); Annett.Maderer@unimedizin-mainz.de (A.M.); Peter.Galle@unimedizin-mainz.de (P.R.G.); 2 Institute of Translational Immunology and Research Center for Immune Therapy, University Medical Center Mainz, 55131 Mainz, Germany

**Keywords:** colorectal cancer, p53, systemic therapy, immunotherapy, tumor microenvironment (TME), cancer-associated fibroblasts, signaling, targeted therapy

## Abstract

**Simple Summary:**

Despite remarkable progress being made by preventive medical check-ups in the last decades, colorectal cancer (CRC) remains one of the most frequent and deadliest cancers worldwide. An understanding of the mutational landscape in CRC is needed to develop new mutanome-directed therapies with stronger efficacy and less side-effects than current therapeutic standard regimes. Carcinogenesis in CRC is a multi-gene driven process, where premalignant cells accumulate successively key tumorigenesis-related mutations. Here, inactivation of the tumor suppressor gene p53 is a hallmark event during tumorigenesis. Mutations of p53 impact the prognosis of patients, enabling the use of targeted therapies such as immune therapy. Alterations of p53 affect not only the tumor biology of cancer cells but also the surrounding tumor microenvironment (TME).

**Abstract:**

Colorectal cancer (CRC) is one of the most common and fatal cancers worldwide. The carcinogenesis of CRC is based on a stepwise accumulation of mutations, leading either to an activation of oncogenes or a deactivation of suppressor genes. The loss of genetic stability triggers activation of proto-oncogenes (e.g., *KRAS*) and inactivation of tumor suppression genes, namely *TP53* and *APC*, which together drive the transition from adenoma to adenocarcinoma. On the one hand, p53 mutations confer resistance to classical chemotherapy but, on the other hand, they open the door for immunotherapy, as p53-mutated tumors are rich in neoantigens. Aberrant function of the *TP53* gene product, p53, also affects stromal and non-stromal cells in the tumor microenvironment. Cancer-associated fibroblasts together with other immunosuppressive cells become valuable assets for the tumor by p53-mediated tumor signaling. In this review, we address the manifold implications of p53 mutations in CRC regarding therapy, treatment response and personalized medicine.

## 1. Introduction

According to the latest global cancer data of the World Health Organization (WHO), the global cancer burden increased to 18.1 million new cases and 9.6 million cancer deaths in 2018 [1]. Despite Europe representing only 9% of the world population, it accounts for 23.4% of global cancer cases and 20.3% of cancer-associated deaths. Colorectal cancer (CRC) is one of the most common cancers in women and men, and accounts for 12.6% (242,000 deaths) of cancer-related deaths in Europe [2]. In previous low-risk European countries such as Spain and several countries in eastern Europe, incidences have rapidly increased, which has been linked to dietary changes in relation to western lifestyle with a high calorie diet, rich in animal-derived proteins, especially red and processed meat, wheat products and high sugar consumption, combined with poor physical activity. Beside Europe, the regions of highest incidence of CRC are Australia and New Zealand, while in Japan, South Korea, countries in the middle east and Slovakia, CRC is the most diagnosed cancer among men [3]. Interestingly, all regions of Africa, as well as Southern Asia, have the lowest incidence rates for both cancers between both sexes [3,4]. But there seems to be a link, between incidence rates of CRC and increasing HDI (Human Development Index) in countries undergoing a major developmental transition [3,4].

In addition to diet and poor physical activity, other risk factors are family history of CRC (stronger association for first-degree relatives), inflammatory bowel disease, smoking, excessive alcohol consumption, obesity, and diabetes [5,6,7,8,9,10,11]. On the other hand, established protective factors are physical activity, the use of hormone replacement therapy, aspirin and, to a minor extent, diets rich in fruit, vegetables, cereal fiber and whole grains, dairy products, or fish and statin therapy. However, the most important preventative factor represents routine endoscopic check-ups, namely colonoscopy, with the extraction of precancerous lesions (polypectomy) [12,13,14,15,16,17,18,19].

CRC begins as a benign adenomatous intestinal polyp from epithelial tissue of the colon, which progresses to advanced adenoma with high-grade dysplasia, invasive adenocarcinoma and, ultimately, metastasis to distant organs such as the liver. This stepwise process is also known as a multistep tumorigenesis, with each step thought to be associated with specific genetic alterations in tumor suppressor genes or oncogenes [20,21].

There are distinct mutation patterns in CRC, which also impact disease progression and overall survival (OS). Mutations of the DNA mismatch repair system are frequently observed together with changes to oncogenes and/or tumor suppressor genes such as *KRAS*, *APC*, *PIK3CA* and *TP53* [22]. Among these, *TP53* is a central player as mutations of the encoded p53 protein are found in ~60% of CRCs, with only *APC* mutations (~80%) occurring more frequently [20,23]. Either loss- or gain-of-function (LOF/GOF) mutations of p53 drive tumor development and growth. LOF suspends the tumor-suppressing role of p53, whereas missense-type mutations tend to be associated with GOF, leading to acquisition of oncogenic properties [20]. In this context, p53 mutations may confer resistance to systemic therapy, which has a profound impact on treatment response and outcomes of patients. Understanding the role of p53 and its underlying mechanisms in CRC has significant implications for individualized and other emerging therapies. In this review, we address the role of p53 mutations for tumor cells as well as the tumor microenvironment and present implications for systemic treatments, immunotherapy and p53 targeting therapies in CRC.

### 1.1. The Physiological Role of p53 in CRC

Up to 60% of patients with CRC show somatic mutations of *TP53*, which is associated with poorer clinical outcomes [24]. *TP53* is also called “the guardian one of the genome” as it plays a crucial role for the regulation of the cell cycle and the stability of the genome. It represents one of the best characterized tumor suppressor genes and is located on the short arm of chromosome 17 (17p13.1). The encoded p53 protein consists of 393 amino acids with four functional domains. The centrally located sequence-specific DNA-binding domain (DBD) (amino acid position 101–306) allows binding to DNA and is frequently altered in p53 mutants, hindering its physiological function [25,26].

The p53 protein is rapidly degraded with a half-life of 6–20 min, with the amount of protein in cells primarily determined by its degradation. Under physiological conditions, p53 is degraded by the ubiquitin-mediated proteolysis. The E3 ubiquitin-protein ligase Mdm2 (MDM2) protein is one of the central enzymes to label p53 with ubiquitin, maintaining low expression of p53 under physiological conditions [26,27]. Under cellular stress, *TP53* becomes activated and p53 is overexpressed to induce cell cycle arrest, apoptosis and senescence. The p53 protein activates p21 (WAF1), a member of the cyclin-dependent kinase (CDK) inhibitors, which are involved in the inhibition of transition from G1 to S phase [28]. Direct activation of the Bcl-2 protein, Noxa and PUMA by p53 induces apoptosis. Further, p53 activates caspase-8 pathways through the activation of cell death receptors (e.g., Fas, DR5 or PIDD) [29]. Cellular senescence, where the cell is, for example, unable to divide, is induced by p53-mediated activation of p16, PML and p21 [30]. Moreover, p53 contributes to genome stability and the regulation of cell metabolism by minimizing mutagenic reactive oxygen species (ROS) [31,32]. Besides direct implications for cells, p53 also affects the surrounding microenvironment, controlling angiogenesis, cell migration and invasion, which will be discussed in detail later.

### 1.2. p53 Mutations in CRC

p53 mutations play a critical role in the adenoma–carcinoma transition during tumorigenesis [28,33]. Although it is mechanistically not fully understood, p53 mutations are less frequent in proximal colon tumors (34%), than in distal colorectal tumors (45%) [34]. The genetic mechanisms of *TP53* mutations include frameshift mutations caused by indels (insertions and deletions) or missense mutations, while the last occur more frequently in CRC [35]. In both cases, the outcome is either suppression of tumor suppressor activity due to LOF or GOF, promoting tumor development and growth [26]. p53 mutants retain their ability to form a protein-tetramer, which may therefore be a mixture of mutated and wild-type (wt-) p53 proteins [36]. In such complexes, wt-p53 protein is hindered from binding to its DNA binding site to express tumor suppressive transcripts due to LOF. GOF occurs when p53 mutants promote the expression of oncogenic transcripts. Here, p53 mutants together with transcription factors enhance the expression of tumor promoting transcripts [26,37].

In conclusion, although p53 alterations are based on distinct mutations, they lead to LOF or GOF of p53, which are hallmark events in the multistep tumorigenesis of CRC.

### 1.3. Treatment of CRC

Initiation of treatment is dependent on specific stratification and staging of the tumor by means of TNM and Union for International Cancer Control (UICC) classification systems. In general, CRC can be divided into four different clinical stages (based on UICC I to IV) according to the expansion of the tumor (T), lymph node (N) involvement and peripheral metastases (M). In this regard, it needs to be determined if the tumor is surgically resectable, if patients may benefit from a neo-/adjuvant chemotherapy, chemoradiotherapy (only rectal cancer) or, in the case of an advanced stage, if palliative chemotherapy needs to be initiated. In general, UICC stage I to III represents localized CRC and patients may undergo surgical resection. In stage II and III, adjuvant chemotherapy may be additionally applied in colon cancer [38]. In terms of rectal cancer, either neoadjuvant or adjuvant chemoradiotherapy in addition to surgical resection is initiated [39]. Stage II colon cancer is additionally stratified according to the microsatellite phenotype which aids in treatment decisions. In this context, a microsatellite stable (MSS) phenotype in comparison to high microsatellite instability (MSI-H) has been associated with a worse prognosis and therefore adjuvant chemotherapy is recommended in these patients [40]. Stage IV marks an advanced stage with distant metastases, also termed metastatic CRC (mCRC). In this case, palliative systemic therapy or resection of metastases is necessary. In mCRC, treatment decisions are also based on certain molecular signatures (wildtype or mutated (K)RAS and (B)RAF mutations) and on tumor site (left- or right-sided tumor) [41]. For a more detailed and in-depth discussion on CRC therapy, we refer to current practice guidelines [39,41]. How p53 can affect treatment response to systemic therapy during different clinical stages will be discussed in the following chapters.

## 2. Treatment Response to Systemic Therapy

### 2.1. Chemotherapy

In the treatment of CRC, several chemotherapeutic drug regimens are considered first-line according to a neoadjuvant/adjuvant curative or palliative approach. The base of cytotoxic chemotherapy is formed by fluoropyrimidine (FP), administered either intravenously as 5-Fluoruracil (5-FU) or as an oral application named Capecitabine, with several possible combinations [42]. In this context, FOLFOX, a combination of 5-FU and Oxaliplatin (OX), is considered to be the standard of care in adjuvant settings (stage II or III) or in advanced stages e.g., metastatic colon cancer (stage IV), according to current international guidelines [38,43]. As an alternative, OX can be substituted by Irinotecan, known as FOLFIRI [44]. Although chemotherapy has shown promising results in the therapy of localized CRC in recent years, the five-year survival rate for metastatic disease remains at around 14% [45,46]. Accumulating evidence suggests that depending on the molecular signature of the tumor, drug resistance to chemotherapy occurs, leading to a poor response and, consequently, worse outcomes. Within this context, p53 has been widely studied to serve as a potential biomarker to predict those patients that may benefit most from certain chemotherapeutic regimens. More specifically, mutations of p53 may have profound effects on the sensitivity to cytotoxic chemotherapy which will be discussed in the following sections.

#### 2.1.1. 5-Fluoruracil

**Figure 1:**
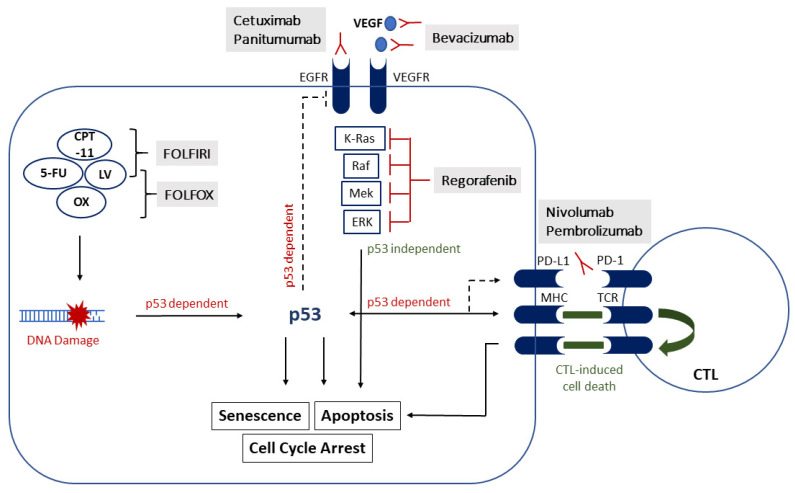
The association of p53 and systemic treatment in colorectal cancer (CRC). Several pathways to induce apoptosis or cell cycle arrest are p53 dependent and; therefore, mutations could possibly affect treatment response. Other pathways seem to be more independent of p53, which provides a potential target in p53 mutated tumors. 5-FU, 5-fluoruracil; CPT-11, Irinotecan; LV, Leucovorin; OX, Oxaliplatin; FOLFOX, combination of 5-FU, LV and OX; FOLFIRI, combination of 5-FU, LV and CPT-11; VEGF, vascular endothelial growth factor; VEGFR, vascular endothelial growth factor receptor; EGFR, epidermal growth factor receptor; PD-L1, programmed cell death 1 ligand 1; PD-1, programmed cell death protein 1; MHC, major histocompatibility complex; TCR, T-cell receptor; CTL, cytotoxic T lymphocyte.

The antineoplastic activity of 5-FU is thought to be partially dependent on p53 to induce cell death and cell cycle arrest as a result of DNA damage (Figure 1) [47]. DNA synthesis is inhibited by 5-FU through its conversion to the active metabolite fluorodeoxyuridine monophosphate (FdUMP). Further conversion of FdUMP into deoxythymidine monophosphate (dTMP) is conducted via thymidylate synthase (TS). Exogenous applied folinic acid, (Leucovorin, LV), inhibits TS and consequently enhances the cytotoxicity of 5-FU, resulting in a greater efficacy in comparison to monotherapy [48]. Whether a loss or an overexpression of p53 contributes to a higher resistance towards 5-FU-based chemotherapy remains unclear. In vitro studies have suggested that a loss of wt-p53 in CRC may confer resistance to chemotherapy, whereas an induction of wt-p53 may, in turn, increase sensitivity to cytotoxic agents [49]. On the other hand, an overexpression of p53 is also correlated with resistance to 5-FU-based chemotherapy [50]. However, earlier studies have correspondingly highlighted the survival benefit of patients with tumors harboring wt-p53 receiving 5-FU in comparison to mutant p53 [51]. Certain types of mutant p53 with a GOF can increase dUTPase expression, which lowers the sensitivity to 5-FU [52]. However, patients with stage III CRC overexpressing p53 and treated with FOLFOX had an improved disease-free survival (DFS) independent of the microsatellite instability (MSI) phenotype [53]. It is currently assumed that CRC cell lines lacking functional p53 are more resistant to 5-FU and OX [54,55]. The underlying mechanism of resistance in p53-depleted colon cancer cells might be due to a lack of ability to bind apoptosis inducing signaling molecules and, hence, malignant cells are protected from cell death (Figure 1) [56]. Therefore, it was expected that the efficacy of 5-FU and OX could be increased by restoring p53 or enhancing DNA damage signaling pathways. In this regard, RITA, a small molecule reactivating p53 and inducing tumor apoptosis, is known to reduce degradation of wt-p53 and to activate its transcriptional function [57]. However, Wiegering et al. showed that RITA enhanced chemosensitivity to 5-FU and OX independent of p53 protein status in several CRC cell lines [58]. Upregulation of a mucin, MUC5AC, conferred resistance to 5-FU through down-regulation of p53 and p21, whereas a knockout increased sensitivity to both, 5-FU and OX, respectively [59]. Combination of Pevonedistat (MLN4924, selective NEDD8 inhibitor) with chemotherapy was able to enhance the apoptotic effects of this therapy in a p53-independent manner in cells harboring both, wt- and mutant-p53 [60]. Furthermore, treatment with dichloroacetate (DCA) re-sensitized CRC cells to 5-FU treatment in previously resistant cells [61].

Amongst others, PUMA, a downstream target of p53 to initiate endogenous apoptosis, becomes activated by 5-FU [62]. In this regard, Huang et al. were able to show that chemoresistance in mutant p53 cells is because of a LOF mutation to activate downstream PUMA-transcription [63]. Another in vitro study showed that β-element reversed the resistance of p53 null colon cancer cell lines by inducing pro-death autophagy and cyclin D3-dependent cell cycle arrest [64]. More recently, in 655 patients with stage III or high-risk stage II CRC treated with adjuvant FOLFOX after curative resection, p53 was immunohistochemically analyzed and patients divided into different groups according to the expression level. Only the group with a mild expression of p53 was associated with a worse outcome during a follow-up of five years [65]. In other studies, no significant association between survival and the status of *TP53* gene and p53 protein expression after adjuvant chemotherapy was found [66,67]. In a population of patients with stage II/III CRC treated with 5-FU-based adjuvant chemotherapy, *TP53* mutation was not significantly correlated with OS. However, a subset of patients with a double mutation of *PIK3CA* and *TP53* showed a worse OS in univariate and multivariate analysis [68]. In wt-p53 5-FU-resistant HCT-8 cell lines, the Wnt pathway suppresses cell cycle arrest and apoptosis through the inhibition of CHK1-induced p53 phosphorylation and stabilization [69]. Liu et al. identified DNA-directed RNA polymerase II subunit RPB1 (*POLR2A*), localized in close proximity to *TP53*, which is also partially co-deleted in p53 depleted colon cancer. An inhibition of the POLR2A gene product with a conjugated α-amanitin antibody increased the sensitivity of 5-FU and OX in a p53-independent manner [70]. Therefore, chemosensitivity might be re-established by introducing personalized treatments in combination to chemotherapy that work independently of p53.

#### 2.1.2. Oxaliplatin

OX-induced DNA damage also activates p53 signaling for apoptosis and cell cycle arrest (Figure 1) [71]. In particular, CRC cell lines harboring wt-p53 were more sensitive to OX treatment, whereas an inactivation of p53 resulted in an increased resistance to OX in vitro. The cytotoxic effects of OX were shown to be Bax- and p53-dependent, although the LOF mutation of p53 was unable to predict response to OX [72]. Another in vitro analysis also showed that apoptosis in wt-p53 HCT-116 cells is mediated via p53, p21 and Bax in response to OX treatment. By contrast, a LOF of p53 HT-29 colon cancer cells had an impaired sensitivity to OX due to dysregulated phosphorylation of the N-terminus and C-terminus serine residues [73]. Others have highlighted the chemosensitivity to 5-FU and OX in wt-p53 cells due to downregulation of ceramide synthase 5 (CerS5) expression, features which were not seen in p53 null cell lines [74]. A comparison between wt-p53 cells and p53-null cells revealed that OX treatment mediates a differential cellular response in colon cancer cells depending on their p53 status, and that targeting possible downstream molecules of p53 may have beneficial effects in p53-depleted cells [75]. Even wt-p53 can be inhibited by other commonly occurring cellular oncoproteins (i.e., MDM2) [76]. An in vitro study showed an enhanced anti-tumor effect in wt-p53 HCT-116 colon cancer cells following treatment with OX and a small molecule inhibitor of MDM2 [77]. Moreover, transfecting wt-p53/MSI CRC cells with miR-195-5p and miR-497-5p increased the sensitivity to OX [78]. By contrast, in patients with colorectal liver metastasis (CRLM) treated with fluorouracil-based neoadjuvant chemotherapy, those with a LOF mutation of p53 had a worse prognosis in comparison to patients without mutations [79]. Yet, Netter et al. did not detect any significant difference in OS or progression-free survival (PFS) in patients with wt- versus mutated-*TP53* metastatic colon cancer treated with 5-FU, OX or Irinotecan [80].

#### 2.1.3. Irinotecan

Irinotecan is a topoisomerase I inhibitor, which is also commonly used in treating CRC instead of OX as part of the FOLFIRI regimen, together with 5-FU and LV (Figure 1). As previously discussed, wt-p53 may be associated with an overall improved response and outcome in patients receiving chemotherapy. In in-vivo xenograft models of CRC, mutated p53 was also associated with a poor response to Irinotecan independent of the microsatellite instability (MSI) phenotype [81]. Tumors with wt-p53 were more likely to be responsive to Irinotecan in an immunohistochemical analysis of CRC specimens [82]. In a large prospective randomized trial of patients with stage III colon cancer receiving either 5-FU/LV or 5-FU/LV and Irinotecan as adjuvant therapy, p53 status was not a predictor of survival. However, the OS of women was positively affected by p53 mutations, depending on the type of mutation [83].

### 2.2. VEGF- and EGFR-Targeted Therapies

#### 2.2.1. Bevacizumab (Anti-VEGF)

In the treatment of metastatic CRC (mCRC), monoclonal antibodies which target signaling cascades of p53 mutants could boost standard chemotherapy regimens (Figure 1). In this vein, biologicals such as Bevacizumab (anti-VEGF, anti-vascular endothelial growth factor) or Cetuximab and Panitumumab (anti-EGFR, anti-epidermal growth factor receptor) are used in combination with standard regimes (only in wt-RAS mCRC for EGFR antibodies). Both pathways, VEGF and EGFR, have significant implications in promoting tumor angiogenesis and cell growth, and the combination of agents targeting these pathways with chemotherapy can enhance clinical response rates [84]. However, *KRAS* mutational status and the tumor site (right-sided or left-sided) were shown to predict treatment response in patients receiving EGFR-targeted therapy. In right-sided colon cancer specifically, anti-EGFR therapy is currently not recommended [41]. Although in-vitro studies have highlighted higher VEGF expression in colon cells with p53 dysfunction [85], the impact of such mutations still remains unclear. A retrospective analysis of 161 patients with mCRC revealed that anti-VEGF-based therapy was superior in relation to OS compared with anti-EGFR-based therapy in p53 mutated tumors, while there was no difference in wt-p53 tumors [86]. An earlier study did not report a significant association of p53 overexpression or mutations in patients treated with Bevacizumab in addition to Irinotecan, 5-FU and LV with respect to survival [87]. Similar results were seen in patients from the CAIRO2 study (randomized phase III study of Capecitabine, Oxaliplatin and Bevacizumab with or without Cetuximab in first-line advanced colorectal cancer). The retrospective analysis of *KRAS* and *TP53* mutation status in this study cohort revealed no impact on PFS and OS [88]. Bevacizumab is known to induce cellular senescence in CRC cells, which appears to be independent of p53 status [89]. This supports most of the clinical data, that p53 is not predictive of treatment response to anti-VEGF targeted therapies. However, if p53 seems to have no potential impact on treatment with anti-VEGF biologicals, patients may benefit from adding them to chemotherapy regardless of the p53 mutational status.

#### 2.2.2. Cetuximab, Panitumumab (Anti-EGFR)

In patients with mCRC, the use of anti-EGFR antibodies is currently restricted to tumors wild-type for *KRAS* as a consequence of poor outcomes in several clinical studies in patients with tumors harboring *KRAS* mutations [90]. More specifically, EGFR induces a transduction cascade that is reliant on RAS and RAF signaling. In this context, p53 inactivation may be associated with EGFR activation, which could provide a rationale for using EGFR-targeted therapies in these patients (Figure 1) [91]. This is further supported by in vitro studies showing a higher Cetuximab sensitivity in cells with down-regulated p53 [92]. Patients with mCRC harboring mutations in both *APC* and *TP53* showed the highest sensitivity to treatment with Cetuximab, and were associated with an overall favorable outcome [93]. Those patients with tumors harboring wt-*KRAS* and a p53 mutation treated with Cetuximab-based chemotherapy had a longer time to progression (TTP) than those without p53 mutation [94]. This is in line with findings from the FIRE-3 trial showing an improved objective response rate (ORR) in a subset of patients with p53 mutations receiving FOLFIRI and cetuximab [95]. In a larger subset of patients with mutated *TP53* and *KRAS* as well as wt-*BRAF*, treatment with Cetuximab was associated with an increase in PFS [96]. Similar results were seen with the use of Panitumumab in patients with tumor p53 mutations, who showed a better PFS than those without such mutations [97]. Therefore, analyzing p53 in extension to *KRAS*/*BRAF* status may identify new subpopulations that could benefit from anti-EGFR treatment—perhaps also independently of tumor site (right-sided vs. left-sided). Contrary to this assumption, patients treated with chemotherapy and Cetuximab, with no mutations in *TP53* and *KRAS*/*NRAS* and *BRAF* combined, had a significantly longer PFS [98]. In another study, the presence or absence of p53 mutations and clinical activity had no impact in mCRC patients receiving first-line FOLFIRI plus Cetuximab [99].

### 2.3. Tyrosine Kinase Inhibitors

Regorafenib is currently the only multityrosine kinase inhibitor (targeting RAS/RAF/MEK/ERK signaling) approved for the treatment of mCRC as a third- or fourth-line option [43]. The oral application of Regorafenib monotherapy has been shown to prolong OS in a large phase III clinical trial in comparison to placebo in pre-treated patients [100]. Cell culture studies suggested that the sensitivity to Regorafenib is not affected by p53 mutations (Figure 1) [101]. The combination of Regorafenib and 5-FU had synergistic growth inhibitory effects in several CRC cell lines with varying mutations, including p53 [102]. Irrespective of p53 status, Regorafenib treatment resulted in the induction of PUMA and apoptosis through the NF-κB pathway [103]. Similar effects were seen with Regorafenib or 5-FU and Idelalisib, a PI3K inhibitor, that was also able to activate PUMA regardless of p53 status [104]. These results highlight that patients with p53-mutated tumors could possibly benefit from such combinations of Regorafenib and conventional chemotherapy, although clinical data are lacking in this context to date.

## 3. Immunotherapy

### 3.1. Immune Checkpoint Inhibitors

Mutations of p53 can also affect the treatment response to immune checkpoint inhibitors (ICI) (Figure 1). In general, CD8+ T cells or cytotoxic T lymphocytes (CTLs) form a crucial defense against cancer cells. However, cancer cells have established mechanisms to escape this immunosurveillance. More notably, an alteration of programmed cell death ligand 1 (PD-L1) on tumor cells leads to an inhibition of the immune response through binding its receptor, programmed cell death protein 1 (PD-1), on CTLs. An overexpression of PD-L1 is seen in several types of cancers, thus promoting escape from host immunity [105]. In more recent years, ICI, and in particular PD-1 targeted antibodies, have shown significant results in relation to substantially improving tumor therapy and re-activating lymphocytes for immune-mediated killing. Especially Pembrolizumab and Nivolumab, both binding to PD-1 and blocking the interaction with PD-L1, have shown promising results in mCRC that has progressed under chemotherapy [106,107]. The approval of ICI is restricted to tumors carrying a high microsatellite instability (MSI-H) and DNA mismatch repair deficiency (dMMR), whereas no response was seen in microsatellite stable (MSS) tumors [108]. Therefore, MSI-H and dMMR have become the most important biomarkers for the initiation of immunotherapy [109]. 5-FU-based chemotherapy was shown to be less effective in these tumors [110]. MSI-H is associated with higher levels of immune infiltrates and this immune contexture can have prognostic significance [111]. A meta-analysis showed that MSI-H tumors have a better prognosis than MSS tumors [40]. In turn, patients with MSS tumors showed higher incidence of *TP53* mutations in comparison to MSI-H tumors [112]. However, a subset of patients may not respond to ICI which could be associated with an impaired antigen presentation through major histocompatibility complex (MHC) I and other regulating mechanisms involving p53.

It is assumed that wt-p53 is directly involved in antigen presentation via MHC I to CTLs, and the presence of wt-p53 promotes effector CTL-induced tumor cell death (Figure 1) [113]. Zhu et al. showed that TAP1, an integral part of MHC I important for antigen presentation, is highly reliant on p53 in response to DNA damage for proper tumor surveillance [114]. In this regard, p53 is a key determinant to improve the effectiveness of the CD8+ T cell response via the Fas-mediated pathway and CD95 expression [115]. Therefore, tumor cells harboring mutant p53 are deemed resistant to this mediated apoptosis. More intriguingly, Wang et al. were able to show that p53 depleted cells had less MHC I on their cell surface in comparison with wt-p53 colon cancer cells [116]. Furthermore, p53 mutation status could be a negative predictor for treatment response because it was associated with reduced immune cell infiltration and PD-L1 expression [117]. In this context, treatment of colon cancer cells with 5-FU can activate MHC I expression in a p53-dependent manner and increase tumor cell susceptibility to cytotoxic T-lymphocyte-mediated lysis [116,118]. This may have a significant impact on treatment response if 5-FU-based therapy is combined with ICI, although clinical evidence to support this assumption is currently lacking. Yet, chemotherapy has been insufficient to create mutations and neoantigens to elicit an immune response [119].

Nevertheless, PD-L1 expression can also be affected in a p53-mediated pathway (Figure 1). An in vitro analysis by Yoon et al. showed that p53-dependent genotoxic stress can mediate the expression of PD-L1 on cancer cells and; therefore, enables the interaction with T cells to escape from immune surveillance [120]. Furthermore, the loss of wt-p53 can increase several inflammatory cells and CD4+ T-cell infiltration, while limiting the infiltration of potent anti-cancer CD8+ T cells [121]. This would imply that mutant p53 can limit the cell killing effects of CD8+ T cells on tumor cells. Cortez et al. showed that patients with lung cancer that expressed high PD-L1 and low p53 levels had lower survival rates than patients with low PD-L1 and high p53 expression [122].

Another ICI that has been approved for MSI-H and dMMR mCRC is the cytotoxic T-lymphocyte protein 4 (CTLA-4) inhibitor Ipilimumab. Its binding to CTLA-4 can re-activate the immune system to recognize and induce apoptosis in cancer cells. Although the impact of p53 status on treatment with Ipilimumab in CRC remains unknown, p53 mutations had no impact on OS in patients with melanoma [123].

More importantly, only 5% of patients with mCRC show this molecular pattern of MSI-H and dMMR. Therefore, it needs to be determined if the remaining 95% could also possibly benefit from ICI. However, MSS and proficient mismatch repair (pMMR) CRC is often characterized by low numbers of tumor mutations, neoantigens and infiltrating immune effector cells, all contributing to increased resistance mechanisms to ICIs [124].

### 3.2. Individualized Immunotherapy

Somatic mutations of genes can result in altered amino acids and peptides that form so called neoantigens, which are not expressed in healthy host cells, and which may be recognized by the host immune system [125]. They can further be subclassified in tumor-associated antigens (TAAs) and tumor specific antigens (TSAs). The latter one compromises mutated antigens which are exclusively expressed by tumors. The neoantigen load is closely linked to an improved patient survival [126], and this may even be independent of MSI-H or MSS status [23]. A higher neoantigen load favors the infiltration of lymphocytes in general, and more importantly tumor infiltrating lymphocytes (TILs). In this context, Malekzadeh et al. were able to show the immunogenic effects of p53 mutated cancer cells [127]. Another study showed that transfection of mutant p53 epitopes into lung cancer cells resulted in the recognition and effective killing by CTLs [128]. Therefore, adoptive cell transfer therapy (ACT) is under development to stimulate the proliferation of specific T cells to recognize these neoantigens and to eventually eradicate tumor cells [129]. In prostate cancer, *TP53* missense mutations were associated with a higher tumor-infiltrating T-cell density [130]. That mutated p53 and its neoantigens can be exploited for the treatment with ACT, has recently been demonstrated in a patient with mCRC [131]. Another form of ACT, chimeric antigen receptor (CAR) T cell therapy, has recently been introduced into early clinical trials for the treatment of CRC [132]. CAR T cells are also designed to target specific antigens associated with cancer cells to induce cell death [133]. For this purpose, T cells are extracted from the patients’ blood, then they are genetically engineered to express chimeric immunoreceptors (CAR) and re-introduced to recognize and eliminate cancer cells more efficiently. Preclinical studies have shown beneficial effects of combining anti PD-1 antibodies and CAR T cell therapy [134]. However, it needs to be determined in future studies how p53 mutations can generate neoantigens that can be exploited for CAR T cell therapy in CRC specifically.

## 4. The Impact of p53 on Treatment Outcomes of Colorectal Liver Metastasis (CRLM)

Approximately 25% of patients with CRC present with metastatic disease at the time of diagnosis [43]. The majority of these patients usually have colorectal liver metastasis (CRLM), as CRC is known to predominantly metastasize to the liver [46]. In the context of CRLM, *TP53* is also one of the most frequently mutated genes, and a predictor of a shorter OS [135]. Hepatic resection is considered the treatment of choice with five-year survival rates between 35% to 60% [136]. The introduction of effective downsizing regimens with chemotherapy has increased the eligibility of patients for resection [137], and showed an improvement in PFS [138]. However tumor recurrence within 12 months after liver surgery remains high [139]. Specifically *KRAS-* and *BRAF*-wt status have been identified as beneficial prognostic biomarkers regardless of the systemic therapy applied in addition to hepatic resection [140]. In a more recent analysis, concomitant mutations of *RAS* and *TP53* were associated with significantly lower five-year OS in comparison to wt-*TP53* among patients undergoing CRLM resection [141]. On the contrary, earlier studies suggested no impact of p53 mutations on long-term outcomes [140]. Instead of systemic therapy, hepatic arterial infusion (HAI) therapy, a locoregional high-dose liver-directed chemotherapy, can also be applied for patients with initially unresectable liver metastases. In this regard, HAI is applied during surgical laparotomy with an insertion of a catheter into the gastroduodenal artery, which remains the gold standard to date [142]. Although HAI has been associated with improved survival [143], patients with CRLM harboring p53 mutations were more resistant to hepatic arterial chemotherapy with floxuridine (fluorinated pyrimidine) [144]. In this context, patients with a high expression of p53 had a survival benefit [145] and a better treatment response [146], especially after 5-FU treatment via HAI. Moreover, in a comparison of patients with resectable and unresectable liver metastases receiving HAI and systemic therapy, concurrent *RAS*/*RAF* and *TP53* alterations were associated with worse survival in primarily unresectable patients [147]. Despite the administration of chemotherapy, adenoviruses containing a p53 transgene have also been developed and tested in phase I and phase II clinical trials for the treatment of p53-deficient cancers [148]. The HAI administration of such adenoviruses resulted in a higher nuclear p53 protein expression and increased apoptotic pathways in the tissues of CRLM [149]. However, following clinical trials of this approach are still lacking.

## 5. The Role of p53 in the Tumor Microenvironment

**Figure 2:**
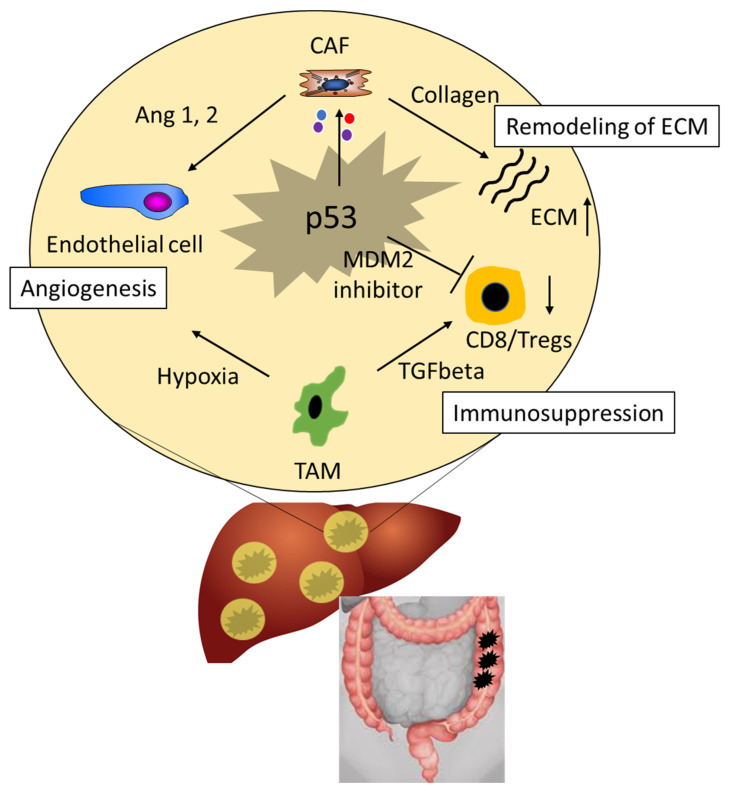
The role of p53 in the tumor microenvironment (TME) of CRC. p53 in tumor cells and surrounding (non-)stromal cells contributes to immune surveillance, angiogenesis and remodeling of extra-cellular matrix (ECM) in CRC. Ang 1, 2, Angiopoietin 1, 2; CAFs, cancer-associated fibroblasts; ECM, extra-cellular matrix; MDM2, E3 ubiquitin-protein ligase Mdm2; TAMs, tumor-associated macrophages; TGF-ß, transforming growth factor beta; Tregs, regulatory T cells.

The tumor microenvironment (TME) increasingly gains the attention of oncologists as it hosts a plethora of immunosuppressive cells, which promote tumor growth and metastasis and counteract immunotherapy. Here, p53 plays a pivotal role not only in tumor cells but also in (non-)stromal cells of the TME (Figure 2) [150]. p53 was found to control tumor immune cell crosstalk as the inhibition of p53 degradation by the MDM2 inhibitor HDM201 increased CD8+ T cells and the CD8/Treg (regulatory T cell) ratio, leading to an improved immune mediated anti-tumor response [151]. The combination of HDM201 with PD-(L)1 blockade further increased the number of complete tumor regressions in a murine tumor xenograft model, suggesting that inhibition of p53 degradation or restoration of p53 might represent an appealing approach for cancer treatment. Besides an immunomodulatory function, p53 mutations dictate the composition of the tumor secretome, which consists of extracellular matrix (ECM) components, remodeling enzymes, exosomes and soluble mediators like growth factors, cytokines and chemokines (Figure 2). Stromal cells are corrupted by these signals and give rise to cancer-associated fibroblasts (CAFs), which are strong allies of the tumor in the TME [152,153,154,155,156,157]. They are the most abundant cell type of the TME and promote multiple aspects of tumor development and growth by three mechanisms: firstly, they remodel the ECM to increase its stiffness and thus inhibit immune cells from infiltrating the tumor stroma [158]; secondly, CAFs stimulate neo-angiogenesis via pro-angiogenic factors (e.g., ang1, 2; angiopoietin 1 and 2), securing the supply of oxygen and nutrients to the tumor [159]; and thirdly, together with tolerogenic cells of the adaptive and inherent immune system, CAFs sustain an immunosuppressive TME, also antagonizing the anti-tumor effect of checkpoint inhibitors [159,160,161,162,163,164]. Although CAFs derive from a variety of cell types, they can arise as a specific phenotype of activated myofibroblasts, which were instrumentalized by tumor mediated signals [159]. Mirroring their mesenchymal heritage, CAFs highly express α-smooth muscle actin (α-SMA), fibroblast activation protein (FAP), type I collagen, platelet derived growth factor receptor-alpha/beta (PDGFRα/β), vimentin, and the cell cycle regulating protein FSP-1 (fibroblast-specific protein, S100A4), which could be exploited for cell-specific drug targeting in the context of anti-stromal therapy [160]. There is rising evidence that the inhibition of p53 in stromal cells, including CAFs, causes immune escape and sustains tumorigenesis [152,165]. p53 is inhibited in stromal cells by onco-miRNA-30d, which is expressed in primarily hypoxic cells in the TME due to tumor triggered hypoxia (Figure 2) [166]. Thus, tumor cells can ablate wt-p53 function in the TME by a non-cell autonomous mechanism. In contrast, cancer cells are not affected by miRNA-mediated p53 inhibition because miRNA-30d only has negligible effects on mutated p53, putatively due to its high stability [167,168].

Besides CAFs, tumor-associated macrophages (TAMs), as myeloid derived suppressor cells, contribute to an immunosuppressive microenvironment and support tumor growth as they are a source of immunosuppressive cytokines (e.g., CCL17, CCL18 and CCL22) and tumor promoting growth factors (VEGF-A, TNF-α) [159,169,170,171]. They stimulate the shift of anti-tumor CD8+ T cells towards immunosuppressive regulatory T cells, which favors immune tolerance and thus act as tumor-promoting agents [172]. In CRC, TAMs possess an intricate role, as their distribution towards the tumor might increase or hinder tumor growth [169]. High numbers of CD68+ TAMs in the invasive front were associated with favorable outcome, while other reports indicated that intratumoral CD68+ TAM counts were related with tumor penetration, lymph node metastasis and advanced stages of colorectal cancer [173,174,175]. Thus, the role of TAMs and their subsets remain to be defined. However, p53 was found to regulate cells of the innate immune system including TAMs. Although the underlying mechanism is unclear and needs further investigation, p53 expression in tumor cells correlated with CD204+ TAMs and density of tumor vessels in CRC [173].

Taken together, there is rising evidence that p53 in stromal and non-stromal cells contributes to immune surveillance of CRC in the TME and aberrant function of p53 promotes tumor growth.

## 6. p53 as a Druggable Target

The therapeutic targeting of mutated p53 is a promising concept in the treatment of CRC and proved to be effective in preclinical models. Therefore, pharmaceutical companies pursued two strategies: Firstly, they sought to develop small molecule inhibitors to directly address p53 mutants and, secondly, they aimed to target pathways which are corrupted by p53 mutants.

Restoration of wt-p53 can be achieved by cysteine-binding compounds (e.g., CP-31398, PRIMA-1, APR-246) [176,177]. Nascent mutant p53 can be refolded by these compounds to its wt-conformation. APR-246, a methylated analogue of PRIMA-1, binds to the p53 core domain, primarily via cysteine mediated binding, and enhances the thermostability of mutated p53, which promotes refolding of mutated p53 to p53 wt-conformation [177,178]. APR-246 is tested in several phase II clinical trials together with carboplatin combination chemotherapy in patients with serous ovarian cancer with mutated p53 (NCT02098343), a combination of APR-246 with azacytidine in p53 mutant myeloid neoplasms (NCT03072043) and a combination of APR-246 with 5-FU and cisplatin in esophageal cancer (NCT02999893) [179].

Besides cysteine-binding compounds, small molecule drugs, such as PK083 and PK7088, bind specifically to the surface cavity of the Y220C p53 mutant and induce refolding to p53 wt-conformation [180,181].

Proper folding of wt-p53 requires zinc as a cofactor, while mutants have a lower affinity to bind zinc. The addition of zinc to cells has been shown to restore the ability of p53 mutants to bind zinc, preventing tumor progression [182]. In this vein, the zinc metallochaperone-1 (ZMC-1), also named as NSC319726, was discovered by screening of the NCI-60 tumor cell line panel and was found to restore the proper folding and transcriptional activity of p53 mutants [182]. ZMC-1 has been shown to induce apoptosis in murine tumor cells of xenograft models, which carry the specific p53 mutation R172H; a mutation, which also exists in human tumors, designated as R175H [183]. In addition, COTI-2, another Zn2+-chelating compound, restores the folding and function of p53 mutants, inhibiting the PI3K–AKT pathway. This leads to tumor cell death and prevents tumor growth in murine xenografts. While the exact mechanism is still under investigation, COTI-2 has already been tested for gynecological and head and neck cancers in phase I clinical trials [26,184]. Furthermore, the small molecule drug, P53R3, has been shown to restore the DNA-binding ability of specific p53 mutants such as 175H, R273H and M237 [185].

While most of the investigational drugs seek to stabilize the wild-type conformation of p53 or enhance the binding to the DNA target site, SCH529074 binds to the p53 mutant’s core domain and increases p53 target gene expression. Additionally, SCH529074 inhibits degradation of p53 by reducing MDM2 mediated p53 ubiquitination [186].

Beside restoring the function and wt-conformation of p53 mutants, other therapeutic strategies seek to deplete or enhance the degradation of p53 mutants. Schulz-Heddergott et al. demonstrated that the heat shock protein (Hsp) inhibitor Hsp90 abrogates Jak2/Stat3 signaling in mouse CRC models by depletion of mutated p53 (R248Q allele), which suppressed tumor growth [187]. Ganetespib, another highly potent Hsp90 inhibitor, was tested in a clinical trial for non-small lung cancer but the drug was found to be futile when compared with standard regimes and the trial was stopped after the first interim analysis [188]. Furthermore, the histone deacetylase (HDAC) inhibitor SAHA degrades mutated p53 by inhibiting HDAC6 and disrupting the HDAC6-HSP90-p53 mutant axis, which is specifically activated in p53 mutants [189].

The repurposing of established drugs is an economically interesting concept that can help to reduce substantial costs in drug development and to bypass the slow pace of new drug discovery. Here, in the clinic already used and so de-risked compounds may be reproposed to treat diseases other than those they were originally approved for [190]. Statins are well known drugs with a favorable safety profile and are applied to treat patients with hypercholesterolemia disease. Statins reduce HMG-CoA (3-hydroxy-3-methylglutaryl coenzyme A) reductase, which is a central enzyme in lipid metabolism. In addition, HMG-CoA controls prenylation/lipidation of proteins. Prenylation is central for different cellular processes like adhesion, migration, and proliferation signaling [26]. p53 binds to sterol regulatory element-binding protein (SREBP) and leads to prenylation of oncogenic proteins in breast cancer [191]. Additionally, statins suppress the Rho GTPases’ prenylation, which promotes nuclear localization and activation of the YAP/TAZ in tumor development [192]. Thus, statins can interrupt these mechanisms, leading to an anti-tumor effect in breast cancers.

Another appealing concept is the use of small interfering RNA (siRNA) to deplete p53 mutants, especially for liver metastasis. The use of siRNA allows for the therapeutic knockdown of virtually any gene (also specific p53 mutants) without affecting other non-targeted genes. Since siRNA offers poor pharmacokinetics and stability in the blood stream, nanoparticles are the ideal vehicles to transport siRNA to liver. We have developed nanohydrogel particles as siRNA carriers, which are therapeutic gene knockdown in liver fibrotic mice [193,194]. Surface modified nanocarriers with target cell specific ligands can also be applied to cell-specific siRNA delivery to cancer cells. Therefore, nanohydrogel particles were coated with mannose for cell-specific siRNA delivery to hepatic M2 polarized macrophages, sharing characteristics of TAM [195,196].

Recently, Han-Chung Hsiue E. et al. published an interesting study where the most common *TP53* mutant R175H (in which arginine at position 175 is replaced with histidine) was targeted with a bispecific antibody (H2) [197]. The highly specific antibody binds to human leukocyte antigen-A (HLA-A) allele on the cell surface, which presents peptide fragments of the *TP53^R175H^* mutant, while its other domain binds to a T-cell receptor to trigger an antitumor response. The bispecific antibody proved to be effective in preclinical models and induced regression of human xenograft tumors in mice, both in early and established tumors.

In summary, there are promising drug candidates for targeting p53 mutants in CRC under development, while only a few drugs are already tested in clinical trials or have shown therapeutical benefit in the clinic. One of the major hurdles is that there is no drug to target all p53 mutants. In the context of personalized medicine, the specific p53 mutations of each patient differs, hence there is need for personalized assessment in order to choose the appropriate combinatorial regimes for targeting p53 mutants together with standard drugs to treat patients with CRC.

A list of relevant drugs targeting p53 mutants in CRC is shown in Table 1.

## 7. Conclusions and Outlook

Despite significant progress in screening programs and therapy in recent years, CRC remains the second most common cancer with an increasing prevalence worldwide. Alterations of the tumor suppressor gene *TP53* contribute to the development of CRC and it is one of the most frequently mutated genes in this context. Besides its oncogenicity, such mutations significantly impact the treatment response to systemic drugs, and, more importantly, the outcome of patients. Standard chemotherapies mostly require wild-type function of p53 to induce apoptosis and cell cycle arrest of tumor cells and; therefore, mutations are thought to confer resistance and decrease chemosensitivity in most cases. Recently, therapeutic approaches have sought to address other targets, circumventing p53 dependent pathways, or possibly exploiting p53 mutations to sensitize tumor cells for chemotherapy. The addition of VEGF- and EGFR-targeted antibodies to standard chemotherapeutic regimens have shown a benefit in a subset of patients, which could be also attributed to p53 independent pathways. In this vein, tyrosine kinase inhibitors like Regorafenib were found to be effective in p53 mutated tumors, but convincing clinical data are still missing to recommend them for therapy at an earlier stage. Besides for the tumor itself, p53 mutations have implications for the surrounding tumor microenvironment. Both immune and stromal cells are compromised by p53-mediated tumor signaling, becoming supportive allies to enhance tumor development and growth. Inhibition of p53 in stromal cells drives the establishment of cancer-associated fibroblasts which shape the ideal nest of the tumor and together with other immunosuppressive cells, counteract immune surveillance. Here, myeloid derived suppressor cells, namely tumor-associated macrophages, possess a pivotal role in the orchestration of immunosuppression. They inhibit anti-tumor T cells (CD8+) and foster their polarization towards an immune tolerant phenotype (regulatory T cells). Thus, p53 mutations re-educate the tumor microenvironment towards an immunosuppressive state.

Although mutations of p53 have been mostly associated with a poor outcome and a worse survival, they offer new therapeutic options when it comes to immunotherapies. As p53 maintains the stability of the genome, p53 mutated tumors are rich in neoantigens which can be exploited for checkpoint-based treatments in personalized therapy. Further, p53 mutated tumors are often compromised in self-antigen presentation, which impairs immune based therapies. Restoration of p53 function can lead to improved immune surveillance by the innate and adaptive immune system and thus enhance the efficacy of checkpoint inhibitors.

However, therapeutic targeting of p53 is difficult as different mutations of p53 exist and drugs need to be combined to tackle this problem in the context of personalized medicine. Besides depletion of p53 mutants, small molecule drugs proved to be effective to restore wild-type conformation and the DNA-binding ability of p53 to induce an anti-tumor effect. Although some molecules were tested in clinical trials, none of them were approved to support established regimes. However, maybe it is not necessary to re-invent the wheel, as repurposing of known drugs could speed-up the translation into clinics. Statins inhibit pathways associated with p53 mutants and have been shown to be effective in the treatment of breast cancer. Novel classes of drugs like RNA-based drugs (siRNA) bear a tremendous therapeutic potential, not only in oncology, and could also be used to treat p53 mutated tumors. However, due to poor pharmacokinetics and low stability in the body, nucleic acids need to be packed in nanocarriers to arrive at their site of action, the cytoplasm of the target cell.

Overall, p53 mutations in CRC have an impact not only for the tumor cells themselves but also for tumor microenvironment, therapy and treatment response.

## Figures and Tables

**Table 1:** List of drugs targeting p53 mutants in CRC.

	Drug	Type of Drug	Mechanism	Stage of Development	Reference
1.	APR-246	Small molecule	Restores wild-type conformation	Clinical phase II	[177,178,179]
	CP-31398	Small molecule	Restores wild-type conformation	Preclinical	[26,180]
2.	PK083	Small molecule	Restores wild-type conformation	Preclinical	[26,180]
3.	PK7088	Small molecule	Restores wild-type conformation	Preclinical	[26,184]
4.	Zinc	Cofactor	Restores wild-type conformation	Preclinical	[26]
5.	ZMC-1	Zn2+-chelating compounds	Restores wild-type conformation	Preclinical	[183]
6.	COTI-2	Zn2+-chelating compounds	Restores wild-type conformation	Clinical phase I	[184]
7.	P53R3	Small molecule	Restores DNA-binding ability	Preclinical	[26]
8.	SCH529074	Small molecule	Restores DNA-binding ability and prevents degradation of p53	Preclinical	[186]
9.	Hsp90 inhibitor	Small molecule	Depletion of p53 mutants	Preclinical	[187]
10.	Ganetespib (potent Hsp90 inhibitor)	Small molecule	Depletion of p53 mutants	Discontinuation of clinical phase I	[188]
	SAHA	Small molecule	Depletion of p53 mutants	Preclinical	[189]
11.	Statins	Small molecule	Inhibition of p53 mutant related downstream targets	Preclinical	[191,192]
12.	siRNA	RNA based therapy	Specific knockdown of p53 mutants	No data yet available	
13.	H2	Bispecific antibody	Specific targeting of *TP53*^R175H^	Preclinical	[197]

## Abbreviations

5-FU	5-Fluoruracil
Ang	Angiopoietin
CAFs	Cancer-associated fibroblasts
CCL	Chemokine ligand
CDK	Cyclin-dependent kinase
CPT-11	Irinotecan
CRC	Colorectal cancer
CRLM	Colorectal liver metastasis
CTL	Cytotoxic T-lymphocyte
CTLA-4	Cytotoxic T-lymphocyte antigen-4
DBD	DNA-binding domain
DFS	Disease-free survival
dMMR	Deficient mismatch repair
dTMP	Deoxythymidine monophosphate
ECM	Extracellular matrix
EGFR	Epidermal growth factor receptor
FAP	Fibroblast activation protein
FdUMP	Fluorodeoxyuridine monophosphate
FP	Fluoropyrimidine
FSP-1	Fibroblast-specific protein, S100A4
GOF	Gain-of-function
HAI	Hepatic arterial infusion
HDI	Human Development Index
HMG-CoA reductase	3-hydroxy-3-methylglutaryl coenzyme A reductase
Hsp	Heat shock protein
ICI	Immune checkpoint inhibitor
LOF	Loss-of-function
LV	Leucovorin
mCRC	Metastatic CRC
MDM2	E3 ubiquitin-protein ligase Mdm2
MHC I	Major histocompatibility complex
micro RNA	miRNA
MMP	Metalloproteinases
MSI-H	High microsatellite instability
MSS/I	Microsatellite stable/instable
NF-κB	Nuclear factor ‘kappa-light-chain-enhancer’ of activated B-cells
OS	Overall survival
OX	Oxaliplatin
PD-1	Programmed death-1
PDGFR α/β	Platelet derived growth factor receptor-alpha/beta
PD-L1	Programmed death ligand 1
pMMR	Proficient mismatch repair
POLR2A	DNA-directed RNA polymerase II subunit RPB1
PUMA	P53 Upregulated Modulator of Apoptosis
ROS	Reactive oxygen species
siRNA	silencing RNA
SREBP	Sterol regulatory element-binding protein
TAMs	Tumor-associated macrophages
TCR	T-cell receptor
TME	Tumor microenvironment
Tregs	Regulatory T cells
TNF-α	Tumor necrosis factor-alpha
TS	Thymidylate synthase
UICC	Union for International Cancer Control
VEGF	Vascular endothelial growth factor
wt	Wild-type
ZMC-1	Zinc metallochaperone-1
α-SMA	α-smooth muscle actin
